# ZnO nanocrystals derived from organometallic approach: Delineating the role of organic ligand shell on physicochemical properties and nano-specific toxicity

**DOI:** 10.1038/s41598-019-54509-z

**Published:** 2019-12-02

**Authors:** Małgorzata Wolska-Pietkiewicz, Katarzyna Tokarska, Anna Wojewódzka, Katarzyna Wójcik, Elżbieta Chwojnowska, Justyna Grzonka, Piotr J. Cywiński, Michał Chudy, Janusz Lewiński

**Affiliations:** 1 Faculty of Chemistry, Warsaw University of Technology, Noakowskiego 3, 00-664 Warsaw, Poland; 2 Centre for Advanced Materials and Technologies CEZAMAT Warsaw University of Technology, Poleczki 19, 02-822 Warsaw, Poland; 3 Institute of Physical Chemistry, Polish Academy of Sciences, Kasprzaka 44/52, 01-224 Warsaw, Poland; 4 Faculty of Materials Science and Engineering, Warsaw University of Technology, Wołoska 141, 02-507 Warsaw, Poland

**Keywords:** Materials chemistry, Nanotoxicology

## Abstract

The surface organic ligands have profound effect on modulation of different physicochemical parameters as well as toxicological profile of semiconductor nanocrystals (NCs). Zinc oxide (ZnO) is one of the most versatile semiconductor material with multifarious potential applications and systematic approach to in-depth understand the interplay between ZnO NCs surface chemistry along with physicochemical properties and their nano-specific toxicity is indispensable for development of ZnO NCs-based devices and biomedical applications. To this end, we have used recently developed the one-pot self-supporting organometallic (OSSOM) approach as a model platform to synthesize a series of ZnO NCs coated with three different alkoxyacetate ligands with varying the ether tail length which simultaneously act as miniPEG prototypes. The ligand coating influence on ZnO NCs physicochemical properties including the inorganic core size, the hydrodynamic diameter, surface charge, photoluminescence (quantum yield and decay time) and ZnO NCs biological activity toward lung cells was thoroughly investigated. The resulting ZnO NCs with average core diameter of 4-5 nm and the hydrodynamic diameter of 8-13 nm exhibit high photoluminescence quantum yield reaching 33% and a dramatic slowing down of charge recombination up to 2.4 µs, which is virtually unaffected by the ligand’s character. Nano-specific ZnO NCs-induced cytotoxicity was tested using MTT assay with normal (MRC-5) and cancer (A549) human lung cell lines. Noticeably, no negative effect has been observed up to the NCs concentration of 10 µg/mL and essentially very low negative toxicological impact could be noticed at higher concentrations. In the latter case, the MTT data analysis indicate that there is a subtle interconnection between inorganic core-organic shell dimensions and toxicological profile of ZnO NCs (strikingly, the NCs coated by the carboxylate bearing a medium ether chain length exhibit the lowest toxicity level). The results demonstrate that, when fully optimized, our organometallic self-supporting approach can be a highly promising method to obtain high-quality and bio-stable ligand-coated ZnO NCs.

## Introduction

**Figure 1:**
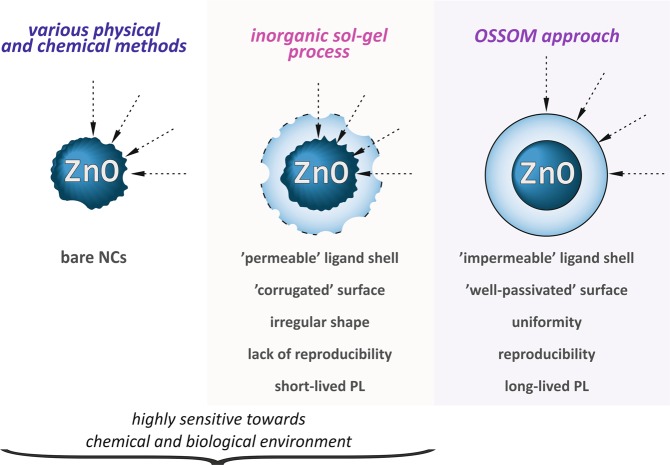
Schematic representation of nanocrystal-ligand interface structure of ZnO NCs prepared by (**a**) different physical and chemical methods, (**b**) the classical sol-gel process and (**c**) the OSSOM approach.

Zinc oxide (ZnO) is one of the most versatile semiconductor material with multifarious potential applications and it has been identified as the fifth most widely used material in the consumer products according to “The Nanotechnology Consumer Products Inventory”^1^. Thus, increasing production of a variety of ZnO nanostructures and associated risk of human exposure to these nanomaterials have prompted the need for a reliable evaluation and a detailed understanding of their potential toxicity, which should be no doubt followed by conscious development of safer-by-design strategies^2^ and bio-oriented nanoarchitectonics concepts^3,4^. The U.S. Food and Drug Administration (FDA) considers ZnO as a “generally recognized as safe” (GRAS) substance, but such indication most commonly refers to the non-toxicity and low chemical reactivity of a bulk material. However, when reduced to the nanosize, ZnO like other nanomaterials^5,6^, can exhibit not only the unique physicochemical properties^7–9^, but also have a potential to generate various and unwanted modes of toxic action including cytotoxic, inflammatory and genotoxic effects^10–12^. The ZnO-induced toxicity might be partly associated with the reactive oxygen species (ROS) generation and/or the release of Zn^2+^ ions^13^, which to a certain extent could be triggered by the interfacial interactions at ZnO nano-bio interface^14,15^. There is a growing consensus about nano-specific toxicity of ZnO nanocrystals (NCs) to be strongly dependent on their physicochemical features (i.e. adverse biological impacts are correlated to size, shape, effective surface charge, character of used ligand molecules and nanocrystal-ligand interface properties) determined mainly by the synthetic procedure and/or is conditioned by exposure conditions^11,12,16^. Remarkably, our recent and comprehensive literature data analysis demonstrated that this relationship is complex and difficult to follow, mainly due to the encountered widely different character of tested ZnO-based nanomaterials in their level of homogeneity and perfection, and/or their incomplete characterization^17^. Thus, despite a number of *in vitro* studies the toxicological impact of ZnO NCs still remains unclear^18^. For example, bare ZnO NCs are very often used in the toxicity screening, however due to their inherently unstable and sensitive surface (Fig. 1) these nanostructures should be *per se* considered as highly hazardous and sensitive toward biological environment. Peculiarly, the interfacial interactions at ZnO nano-bio interface and extracellular environment promote dissolution and the release of potentially toxic cations into the cellular environment^13–15^. This perception has been also supported by several theoretical reports, which considered the uncoated ZnO NCs as one of the most toxic nanoparticles made from metal oxides^19–21^. In turn, toxicity posed by bare ZnO NCs may be controlled and reduced to a certain level by the surface chemistry, e.g. surface doping^22,23^, surface passivation by distinct inorganic material^14,24,25^ or external protective ligand-coating strategies^10,26,27^. Another important parameter that determines cellular contact and particle uptake, and has a significant effect on nanomaterials toxicity is the surface charge of NCs, typically expressed as the zeta potential. Some recent scientific reports indicate that positively charged NCs usually penetrate cells more readily^28–30^ or induce higher endothelial cells leakiness^31,32^. Nevertheless, both systematic studies on toxicological profiles of surface-modified ZnO NCs and the nano-specific toxicity defined by multiparametric structure-property relationship remain highly challenging^33^. Most of published toxicological studies concern ZnO nanostructured materials derived from inorganic sol-gel procedures^11,12,17^, which provide ZnO NCs with highly corrugated surface structure and coated with ‘swollen’ organic ligand shell (Fig. 1)^34,35^. This type of superficies is often associated with the instability and permeability of the organic coating towards both chemical^35,36^ and biological environment^17^, which induce the higher core accessibility as well as the faster core dissolution. Therefore, the lack of high-quality NCs may be identified as one of the limiting factors for development of ZnO NCs-based devices and bio-applications. More recently, organometallic approaches have emerged as a very advantageous alternative to the omnipresent traditional sol-gel route toward quantum-sized ZnO crystals with outstanding properties for advanced applications. For example, Chaudret and Kahn have developed a method for the preparation of ZnO NCs, utilizing R_2_Zn as the organometallic precursor under ambient conditions and in the presence of long-chain amines acting as L-type ligands^37,38^. In this case the resulting amine-stabilizing layers exhibit the ‘dynamic surface structures’ due to relatively weak interactions between the inorganic core and the L-type coating ligands^39,40^. In order to avoid such unfavorable effects associated with ZnO NCs derived from the classical sol-gel synthesis or the aforementioned organometallic approach (e.g. the highly active and unstable surface or permeability of the organic shell), only very recently, we have elaborated a general and convenient **o**ne-pot **s**elf-**s**upporting **o**rgano**m**etallic (OSSOM) approach based on the controlled exposition of a [RZn(X)]-type precursor (X = monoanionic organic ligand) to air leading to an exquisite variety of colloidal quantum-sized ZnO crystals coated with an strongly anchored X-type organic ligand shell^35,36,41–45^. In this case, strongly bonded organic ligands provide stability and effective surface passivation to the resulting NCs (Fig. 1). The superiority of newly elaborated OSSOM approach over the commonly used sol-gel process for the preparation of colloidal ZnO NCs and vastly different surface-ligand interfaces of the respective NCs were well resolved by our very recent advanced comparative characterization through dynamic nuclear polarization (DNP-)enhanced solid-state nuclear magnetic resonance^35^. These results nicely collaborate with our earlier observation that the OSSOM method provides colloidal ZnO NCs well-protected by an impermeable layer of organic ligands^36^, which is prone to self-assemble or further processability^41,42^. For example, the impermeability of the organic shell of these NCs allows for effective functionalization by standard Cu(I)-catalyzed click chemistry with retaining the photoluminescence properties of ZnO NCs^36,45^ (strikingly, the sol-gel process affords the NCs with insufficiently capped surfaces and thus inadequate for post-synthetic functionalization mediated by copper catalysts^36^). Moreover, the unprecedented high-quality of these NCs is also well-documented by the observed ultra-long-lived electron-hole separation (up to 2.2 μs)^17,46^, which likely results from exceptional nanocrystal-ligand interface, as well as the EPR silence under standard conditions^17^. Thereby, all these properties make ZnO NCs engineered by the OSSOM method prospective for a multitude of applications, including photocatalysis^46–48^ and biomedical applications^17^.

Coating the surface of NCs with FDA-approved polyethylene glycols (PEGs) is one of the most commonly used strategy to provide stealth characteristic of NCs and to improve nanomaterials biocompatibility, long-term stability and solubility in biological fluids^49–52^. However, the PEG corona must provide a sufficiently thick layer to shield the NC surface effectively from the interaction with biological environment and from undesirable aggregation. Therefore, there is continuous interest to investigate a direct PEGylation protocols and the selection of proper PEG-type protective coating as well as the effects of individual parameters on the behavior of PEGylated NCs. Advancing the OSSOM method, we have turned our attention to quantum-sized ZnO NCs passivated by alkoxyacetate ligands (acting as miniPEG prototypes based on the analogy to the single building block of the PEG) as a model system for *in vitro* cytotoxicity studies. Our preliminary studies demonstrated that colloidal ZnO NCs with the core sizes of 4–5 nm and densely packed brush-type shell of 2-(2-methoxyethoxy)acetate ligands exhibited negligible negative impact on the selected lung cell lines^17^. This observation was in strong contrast to the widespread and not scientifically proven information about the toxicity of quantum-sized ZnO crystals which are considered to be the most toxic due to the possible direct penetration of the cell membrane^6^. Simultaneously, we also indicated that the resulting NCs possess well-passivated surface, which prevents both leaching of Zn^2+^ ions from the inorganic core and the ROS generation^17^. Encouraged by these results, we sought to investigate systematically nano-specific toxicity of ZnO NCs capped by different miniPEG-type ligands. Thus, we wondered how minor changes in the backbone of alkoxyacetate ligands will affect physicochemical properties and nano-specific toxicity defined by structure-property relationship of ligand coated ZnO NCs prepared *via* the OSSOM method. Towards this aim, we report on (*i*) the preparation of colloidal and bio-stable ZnO NCs coated by alkoxyacetate ligands with different tail length and strongly anchoring the carboxylate head-group along with the influence of applied coating on (*ii*) the NC’s properties including the inorganic core size and the hydrodynamic diameter, solution-stability as well as optical parameters, i.e. photoluminescence (PL) quantum yield and PL decay times, and (*iii*) their biological activity toward lung cells. The toxicological effect of ZnO NCs on normal human (MRC-5) and cancer human (A549) lung cells was evaluated by determination and comparison of the cell metabolic activity at different NCs doses, ability to ROS generation and corresponding cell death mechanism.

## Results and Discussion

### Preparation and characterization of colloidal ZnO NCs coated with selected alkoxyacetate ligands acting as miniPEG prototypes

**Figure 2:**
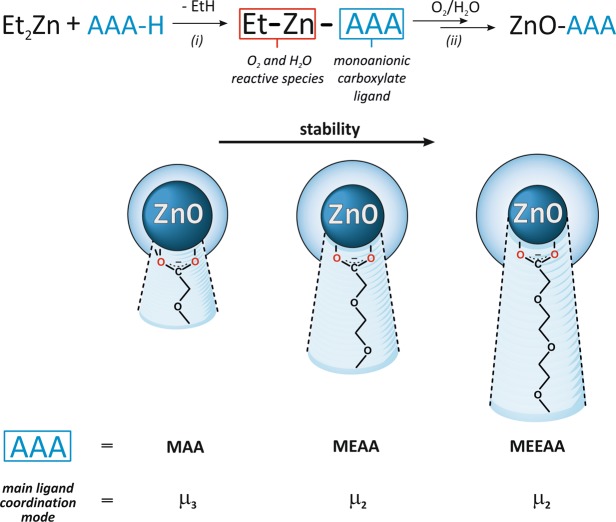
Schematic representation of the synthesis of ligand-coated ZnO NCs from [EtZn(AAA)]-type organometallic precursors and the effect of the ligand’s character on its surface binding mode and ZnO NCs stability.

The OSSOM approach was used as a model platform to synthesize a series of ZnO NCs coated with alkoxyacetate ligands as miniPEG prototypes. In order to verify the effect of the supporting ligand (the length of its ether tail) on ZnO NCs’ physicochemical properties and biological activity toward lung cells, three commercially available alkoxyacetic acids (AAA-H) were selected as pro-ligands for the preparation of ZnO NCs coated with the monoanionic AAA ligands that act as native capping agents in the two-step OSSOM method, i.e. methoxyacetic acid (MAA-H), previously applied 2-(2-methoxyethoxy)acetic acid (MEAA-H)^17^ and 2-[2-(2-methoxyethoxy)ethoxy)acetic acid (MEEAA-H) (Fig. 2). The first step involved the synthesis of an appropriate [EtZn(AAA)]-type precursor in an equimolar reaction between commercially available Et_2_Zn and the corresponding AAA-H pro-ligand in THF. The *in-situ* synthesized [EtZn(AAA)]_n_ precursors were characterized by H^1^ NMR and infrared spectroscopy (see Experimental Section). We also note that ethylzinc derivative of MAA-H ([EtZn(MAA)]_n_) crystalized from a toluene solution as a hexanuclear complex, and the molecular structure of this complex^53^ as well as few other alkylzinc carboxylates were previously described^54–57^. In the second step, a THF solution of the respective [EtZn(AAA)]_n_ organometallic precursor was exposed to air to initiate transformations leading to ZnO NCs capped with the monoanionic carboxylate moieties. The formation of stable colloidal ZnO NCs was completed after approximately one week. After the one-pot synthetic procedure, the ZnO-AAA NCs were separated from the THF solution using hexane, then washed two times with THF and used for further characterization. Both ZnO-MEAA and ZnO-MEEAA NCs gave stable colloidal solutions in DMSO and THF, while ZnO-MAA NCs gave a colloidal solution only in DMSO and formed a precipitate during storage time in THF. It is worth noting that all of ZnO-AAA NCs initially dispersed in DMSO could be also efficiently transferred from an organic solvent to water or to biological media by gradual dissolution and remained stable for several days.

**Figure 3:**
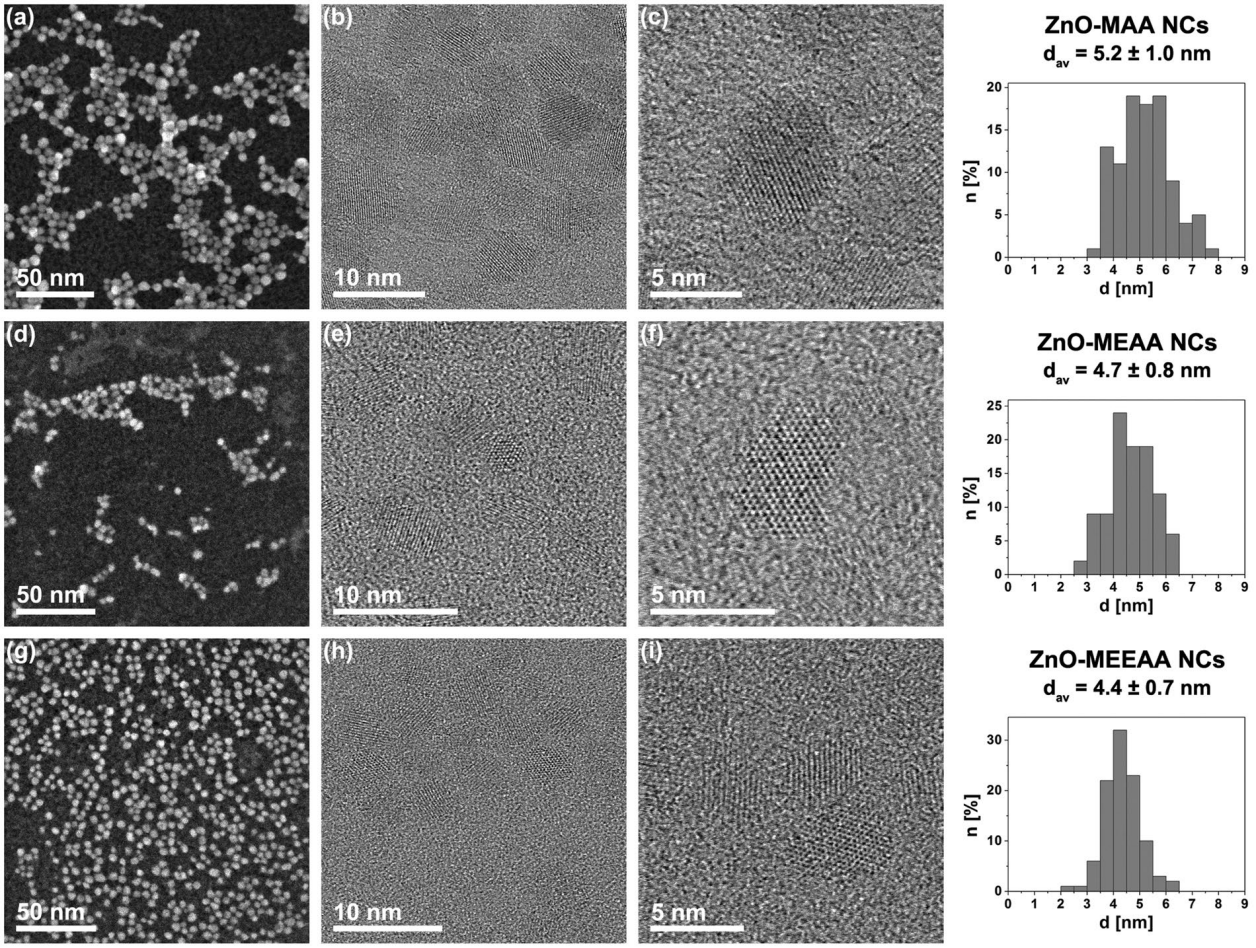
Representative STEM and HRTEM images of (**a**–**c**) ZnO-MAA, (**d**–**f**) ZnO-MEAA, (**g**–**i**) ZnO-MEEAA NCs.

The resulting ZnO-AAA NCs were characterized using several analytical techniques, including Scanning Transmission Electron Microscopy (STEM), Powder X-ray Diffraction (PXRD), Dynamic Light Scattering (DLS), Thermogravimetric Analysis (TGA), Infrared Spectroscopy (FTIR) and UV-Vis absorption as well as steady-state and time-resolved photoluminescence (PL) spectroscopy. The STEM and HR TEM micrographs show well-dispersed, uniform and spherically-shaped ZnO-MAA, ZnO-MEAA and ZnO-MEEAA NCs with a mean core diameter of 5.2 ± 1, 4.7 ± 0.8^17^ and 4.4 ± 0.7 nm, respectively (Fig. 3 and Table 1). It is worth mention that ZnO NCs capped with the alkoxyacetate ligand with the longest ether chain are characterized by the smallest core diameter and the narrowest size distribution (Fig. 3). The phase purity and the degree of crystallinity were confirmed by PXRD measurements (Fig. S1 in the SI). In all cases the observed diffraction patterns corresponded well to that of standard wurtzite- type ZnO and no other crystalline phases could be distinguished. Using DLS we explored the dynamic properties modified through interactions with the suspending medium both in THF and DMSO. Interestingly, the hydrodynamic diameters for the NCs capped with MAA or MEEAA were different when dispersed either in THF or in DMSO (see Table 1). The average hydrodynamic diameter for ZnO-MAA NCs coated with the carboxylate bearing the shortest ether chain was found to be ~11 nm in THF and ~9 nm in DMSO, respectively. For ZnO-MEEAA NCs capped with the alkoxyacete ligand with the longest ether chain the average hydrodynamic diameters were ~11 nm in THF and ~13 nm in DMSO. Only in case of ZnO-MEAA NCs hydrodynamic diameters were found to be around 12 nm for both solvents^17^. This observation indicates various ZnO NCs surface-solvent interactions supported to counteract the aggregation process, which strongly depend on the tail length of capping ligand. Then, the water-stability and the tendency of ZnO NCs to form aggregates were determined by zeta potential measurements. As listed in Table 1, the surface charges of ZnO-MAA, ZnO-MEAA and ZnO-MEEAA NCs dispersed in water were 36.2 ± 1.9 mV, 34 ± 1.1 mV and 30.8 ± 2.9 mV. The resulting ZnO-AAA NCs exhibit a mildly positive zeta potential greater than the critical ± 30 mV^30^ (note that the magnitude of the zeta potential provides information about NCs stability and NCs with zeta potentials more positive than +30 mV or more negative than −30 mV are considered colloidally stable) and thus the NCs are fairly stable in aqueous environment likely due to both the mini-PEG-type coating and the presence of sufficient repulsive forces between individual NCs. Moreover, the observed relatively high surface positive charges could be also associated with low Zn^2+^ ions dissolution from the inorganic core^17,58^. However, the zeta potential values are slightly affected by the size of inorganic core and the thickness of the protecting shell around the NC surface, being smallest for the NCs coated with the carboxylate ligand bearing the longest ether tail, which may result in slightly decreased physical colloidal stability in water. Thus, the presence of a long-chain ligands makes repulsive electrostatic forces weaker compared to attractive van der Waals forces and long-ranged ligand-ligand interactions between the NCs.

**Table 1:** Physical characterization data for ZnO-AAA NCs.

ZnO NCs	Core size [nm]	HD [nm]	Zeta potential^3^ [mV]	Max Abs [nm]	Max PL [nm]	QY [%]
ZnO-MAA	5.2 ± 1^a,2^ 4.49 ± 0.45^b,1^ 4.53 ± 0.44^b,2^	10.97^1^ 8.93^2^	36.2 ± 1.9	332^1^ 333^2^	520^1^ 530^2^	30^1^ 25^2^
ZnO-MEAA^17^	4.7 ± 0.8^a,2^ 3.97 ± 0.50^b,1^ 3.66 ± 0.55^b,2^	12.55^1^ 12.44^2^	34 ± 1.1	328^1^ 325^2^	516^1^ 527^2^	14^1^ 28^2^
ZnO-MEEAA	4.4 ± 0.7^a,2^ 3.95 ± 0.51^b,1^ 3.62 ± 0.55^b,2^	11.35^1^ 12.95^2^	30.8 ± 2.9	329^1^ 324^2^	519^1^ 525^2^	17^1^ 33^2^

Abbreviations: Solvent: ^1^THF, ^2^DMSO; ^3^water; Core size estimated from: ^a^TEM measurements, ^b^optical measurement (see SI), HD – hydrodynamic diameter is given as Z-average size value.

The FTIR spectra of ZnO-AAA NCs (before and after purification) along with FTIR spectra of the organometallic precursors and the parent carboxylic acids are shown in Fig. S2 in the SI. After the purification, the traces of parent AAA-H pro-ligands are no longer observed, which demonstrates that all the carboxylate passivating moieties are deprotonated (Fig. S3 in the SI). Additionally, the strong C=O absorptions are red-shifted compared to the corresponding vibrations for the free acids. In the spectra of ZnO-MEAA and ZnO-MEEAA NCs, the observed two bands of relatively strong intensity, characteristic for the asymmetric ν_as_(COO^−^) and symmetric ν_s_(COO^−^) stretches at 1589 cm^−1^, 1589 cm^−1^ and 1419 cm^−1^, 1417 cm^−1^ (Δν_MEAA_ = 170 cm^−1^, Δν_MEEAA_ = 172 cm^−1^), indicate the bridging bidentate μ_2_ coordination mode of the coating carboxylate moieties. For ZnO-MAA NCs, bands corresponding to ν_as_ and ν_s_ stretches appear at 1601 cm^−1^ and 1439 cm^−1^, respectively. Moreover, the presence of additional bands at 1579 cm^−1^ and 1427 cm^−1^ indicates different coordination modes of the carboxylate groups, μ_2_ bridging or both μ_2_ bridging and μ_3_ chelating, which correlates well with the described previously FTIR spectrum of the molecular precursor [EtZn(MAA)]_6_^53^. Moreover, the FTIR data collected for the NCs capped with MAA, MEAA and MEEAA showed expected characteristic vibration of Zn-O species at 424 cm^−1^, 436 cm^−1^ and 456 cm^−1^, respectively.

Thermal stability of the NCs was also tested and the respective TGA profiles collected under the air atmosphere (with heating rate of 5 °C/min) are shown on Fig. S4 in the SI. For ZnO-MAA NCs one main decomposition step was observed with two overlapping components at 260 °C and 277 °C. The decomposition process was completed with a total weight loss of 20.5%. The TGA profile of ZnO-MEAA NCs exhibited two-step decomposition pathways, comparable to that observed for ZnO-MAA NCs, with their maxima shifted to higher temperatures (280 °C and 335 °C) with the total weight loss of 54.9%. In the TGA profile of ZnO-MEEAA NCs one decomposition step was observed with the maximum decomposition rate at 291 °C. The decomposition process was finished at ca. 440 °C with a total weight loss of 65.1%. These results indicate that the organic shell coating the ZnO NCs is densely packed^17^ and as far as we know, a high-density brush-type arrangements of short-chain PEG molecules is more preferable for achieving optimum stealth properties of NCs in different biological environments^49–52^.

### Photophysical study of ZnO-AAA NCs

A thorough photophysical study was executed for ZnO-AAA NCs in THF and DMSO solutions, and the corresponding UV-Vis and PL emission spectra are shown in Fig. S5 (see the SI). The NCs revealed a broad absorption in the ultraviolet region of 320-340 nm and broad PL emission peaks were centered between 515 nm and 530 nm (Table 1). We noted that the applied solvent virtually did no influence the absorption spectra. On the other hand, the PL emission spectra collected in DMSO, were red shifted ca. 10 nm, when compared with those collected in THF (Table 1). The absolute PL quantum yield (QY) determined in organic solvents was found to be up to 30%, 28%, 33% for ZnO-MAA, ZnO-MEAA and ZnO-MEEAA NCs, respectively. In general, the QY for the studied NCs are comparable with those presented for ZnO NCs synthesized by inorganic methods^59,60^. We also determined the mass attenuation coefficient (µ) for all as-prepared ZnO-AAA NCs. The results are comparable in both tested solvents and strongly depend on the molecular weight of the whole studied systems (see Fig. S6 in the SI).

To determine the long-term stability for synthesized ZnO NCs in THF and DMSO, the spectroscopic shelf-life studies were performed (Fig. S7 in the SI). The colloidal stability of ZnO-AAA NCs was monitored up to 75 days (see Fig. S7 in the SI). The homogeneity of solutions remained virtually unaffected during the storage and no precipitation was observed. Nevertheless, over this time period we noticed a drop in PL emission. The PL emission decreased faster in THF than in DMSO until its stabilized at a certain level (i.e. 53%, 61% and 83% of the initial PL value in THF and 65%, 79% and 95% in DMSO, for ZnO NCs capped with MAA, MEAA and MEEAA, respectively) (Fig. S7 in the SI). Thus, ZnO-AAA NCs were found to be more stable in DMSO solution and the organic solvent-stability generally increased proportionally with increasing the thickness of the protecting shell around NCs (note that the observed water-stability of ZnO-AAA NCs is inversely related to the ligand length, *vide supra*).

Additionally, the PL time-resolved spectroscopy was used to determine PL decay times for ZnO-AAA NCs. Preliminary studies shown that the OSSOM method provides quantum-sized ZnO crystals with ultralong PL decay (up to 2.1 µs)^17,46^, which is several orders of magnitude longer than that observed for ZnO-based nanostructured fabricated using sol-gel method^61–63^. Thus, we decided to investigate the effect of lengthening ether tails of the coating alkoxyacetates on the PL lifetime’s properties. It was found that the ether chain length of AAA ligands has a modest influence on the PL decay time (Fig. S8 and Table S1 in the SI). In each case, it was possible to distinguish four analogous contributions. The major decay contribution was a fast decaying component with decay time around 30 ns (~70%), then a second component around 100-150 ns (7–15%) and third one in the range 700-900 ns (~12%). The fourth decay time was virtually unaffected by the ligand’s character, both in its value (~2.3 µs) and contribution (5–6%). Thus, in contrast to the common wisdom that the charge-carrier separation and transfer processes are controlled by the coating organic ligands^64,65^, the character of organic ligand shell in ZnO NCs engineered by the OSSOM method has essentially minor effect on modulation of different parameters of charge formation and separation, and the corresponding PL decays presumably relate to the unique inorganic core-organic shell interface. We also show that the resulting nanocrystal-ligand interface works as a hole stabilizer, which dramatically slows down charge recombination process. Moreover, the long-lived luminescence is determined rather by the applied synthetic procedure and the resulting unique surface properties. In contrast to the kinetically controlled sol-gel process^63,66–68^ leading to ZnO NCs with an imprecise character of surface structure and relatively fast PL decays^61–63^, the OSSOM procedure likely enables thermodynamic control over the nucleation and growth of NCs, which is associated with the impermeability of organic coating and the unprecedented physicochemical properties of the nanocrystal-ligand interface of ZnO NCs^35,36^.

### Nano-specific toxicity of ZnO-AAA NCs

To date, the main area of nanotoxicology research, crucial for characterization of the potential hazards, was focused on screening and toxicity profiling the vast array of engineered nanomaterials, including also a variety of metal oxides^69–73^. Nevertheless, the unambitious interpretation of experimental data, and particularly, the correlation of the NCs properties to their nano-specific toxicity is still a very challenging issue^74^. To the best of our knowledge, the inherent properties of inorganic-organic interface seem to be one of the most important factors influencing the interactions with biological fluids, critically important for the potential use of ZnO NCs in biology and medicine^75^. Thus, we decided to evaluate the influence of both intrinsic and extrinsic physicochemical properties on the nano-specific ZnO-AAA-induced cytotoxicity. For this purpose, the NCs initially dispersed in DMSO were transferred into cell culture medium by gradual dissolution. It is widely believed that nanostructured materials are harmful mainly for the respiratory system, thus A549 and MRC-5 cell lines were selected as *in-vitro* models for internal malignancies and normal lung cells, respectively. The ZnO NCs-induced cytotoxicity was assessed using cell metabolic activity (MTT) assay. The interference resulted from ZnO-AAA NCs inside and outside cells was eliminated by performing similar experiments using a control (cells untreated with ZnO-AAA NCs). Despite the fact that DMSO is considered as a relatively low toxic solvent and it is widely used as a solvent for pharmacological substances as well as to several other applications including therapeutic applications and experimental *in vitro* studies^76^, we also verified the effect of DMSO on the proliferation and metabolic activity of selected types of lung cells on *in vitro* cultures. As we previously estimated the highest safe value of DMSO solvent concentration in the negative control is equal to 0.25 vol% and it corresponds well to the highest applied ZnO NCs concentration, i.e. 25 μg/mL^17^.

**Figure 4:**
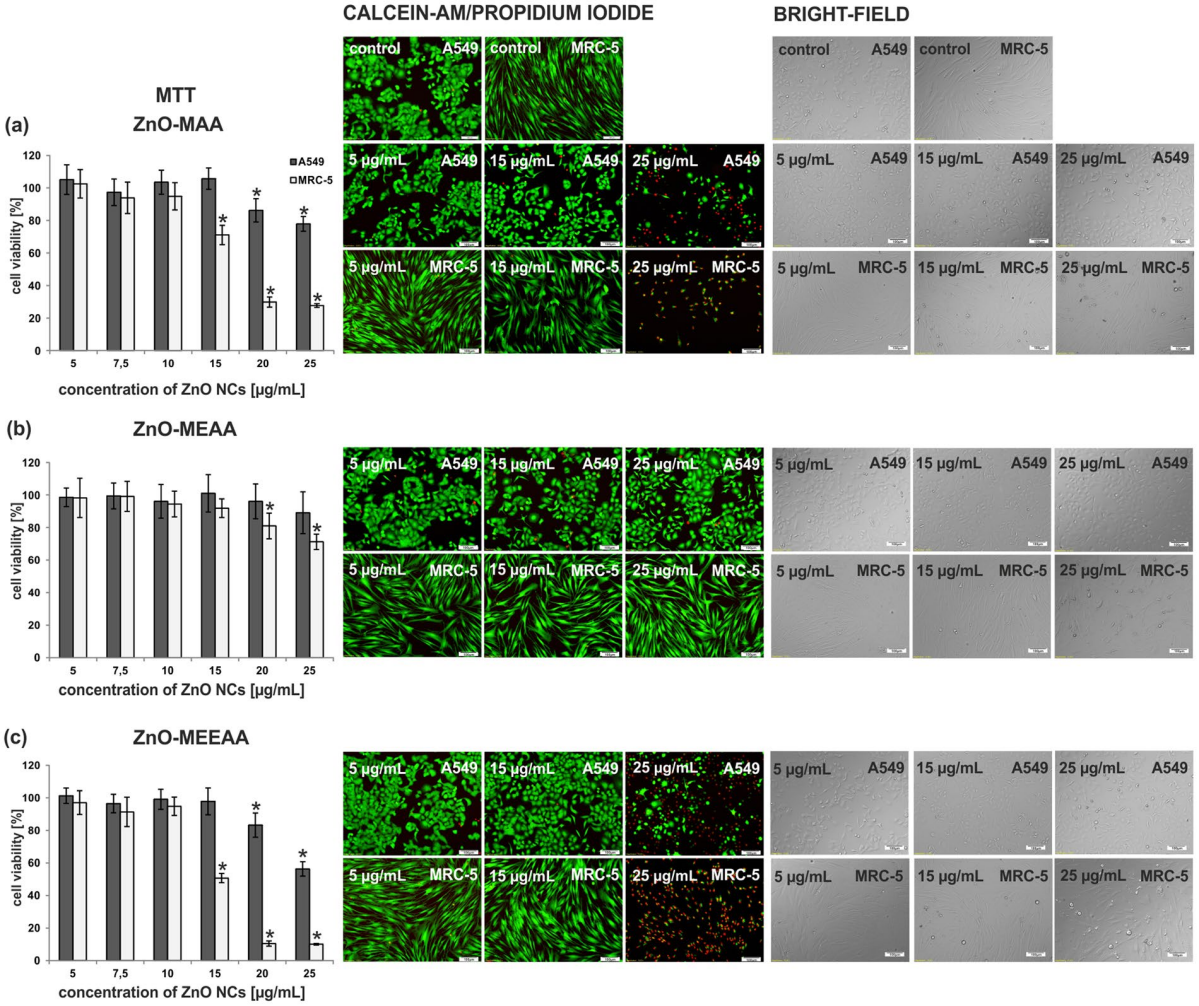
MTT cell viability evaluation and observation of the morphological changes of A549 (cancer) and MRC-5 (normal) cells after 24 h of incubation with NCs: (**a**) ZnO-MAA, (**b**) ZnO-MEAA, (**c**) ZnO-MEEAA. Experimental data were expressed as the mean of cell viability ± standard deviation (SD) of at least four individual experiments with six replicate wells. Asterisks denote statistical significance at p < 0.05.

The influence of ZnO-AAA NCs on MRC-5 and A549 viability cells, determined for 24 h at concentrations between 0 and 25 μg/mL is shown in Fig. 4. All of the studied NCs showed a dose-dependent cytotoxicity and none of them exhibited strong toxicity toward A549 cells. Both, ZnO-MAA and ZnO-MEEAA NCs (i.e. the NCs coated by carboxylates bearing the shortest and the longest ether tail) exhibited a similar toxicological profile toward MRC-5 cells. They were non-cytotoxic for MRC-5 cells at the lowest dose (5–10 µg/mL), while at higher doses (15, 20 and 25 µg/ mL) a significant reduction in viability (i.e. 71%, 30%, 28% and 51%, 10%, 10%, respectively) was observed. Strikingly, only the ZnO-MEAA NCs (coated by carboxylate bearing the medium ether chain length) induced the minor reduction in the viability both for MRC-5 cells and A549 cells even when the highest ZnO NCs concentration (25 μg/mL) was applied^17^. Furthermore, the ZnO-AAA NCs’ cytotoxicity and the changes in cell morphology were observed using an inverted fluorescence microscopic observations of cell cultures (Fig. 4). The cells were stained with Calcein-AM and Propidium Iodide solutions, which stain viable (green fluorescence) and dead cells (red fluorescence), respectively. Generally, the significant cytotoxic effect was observed only for the normal lung cells. This may be due to the fact that normal cells are less resistant to various factors than cancer A549 cells. The MTT data analysis indicate that there is a subtle interconnection between inorganic core-organic shell dimensions and toxicological profile of ZnO-AAA NCs. As we noted above, the geometry factors including the size of inorganic core and the thickness of the protecting shell around the NC surface as well as the effective surface charge and cytotoxicity are affected by the ether tail length of the supporting AAA ligand. Strikingly, only the NCs coated with the MEAA carboxylate bearing the medium ether chain length are both stabile and resistant toward biological environment without generating toxic side effects.

**Figure 5:**
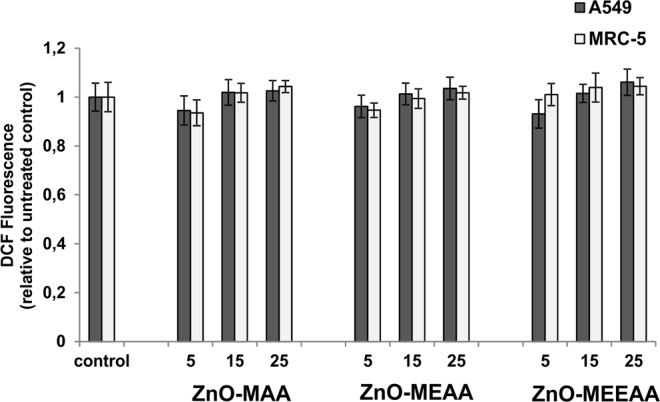
The effect of ZnO NCs on intracellular ROS production. The A549 and MRC-5 cells were treated with 5, 15 and 25 µg/mL ZnO NCs for 24 h prior to the ROS determination including addition of DCFH-DA for 30 min followed by fluorescence measurement. The values are represented as mean ± S.D. of three individual experiments.

In contrast to the common opinion that upon the exposure to ZnO NCs the cellular metabolic activity is often associated with the oxidative stress, we latterly confirmed that ZnO-MEAA NCs do not affect the generation of ROS^17^. In order to verify whether the related NCs with the shorter MAA and the longer MEEAA coating ligand induce ROS production, we introduced a cell-permeable non- fluorescent 2’,7’-dichlorofluorescin diacetate (DCFH-DA) to quantify reactive oxygen species. The experimental data for ROS induction in A549 and MRC-5 cells after their exposure to 5, 15 and 25 µg/mL of ZnO-MAA and ZnO-MEEAA NCs for 24 h along with previous results for ZnO-MEAA NCs^17^ are shown in Fig. 5. A slight shift in DCF fluorescence was found to be dose-dependent, but differences observed in ROS level were negligible (relative for the control tests) for all of the tested ZnO-AAA NCs. (Fig. 5). The results indicate that the cytotoxicity and cell death induced by high concentrations of the ZnO-AAA NCs could not be considered as a ROS-dependent process.

**Figure 6:**
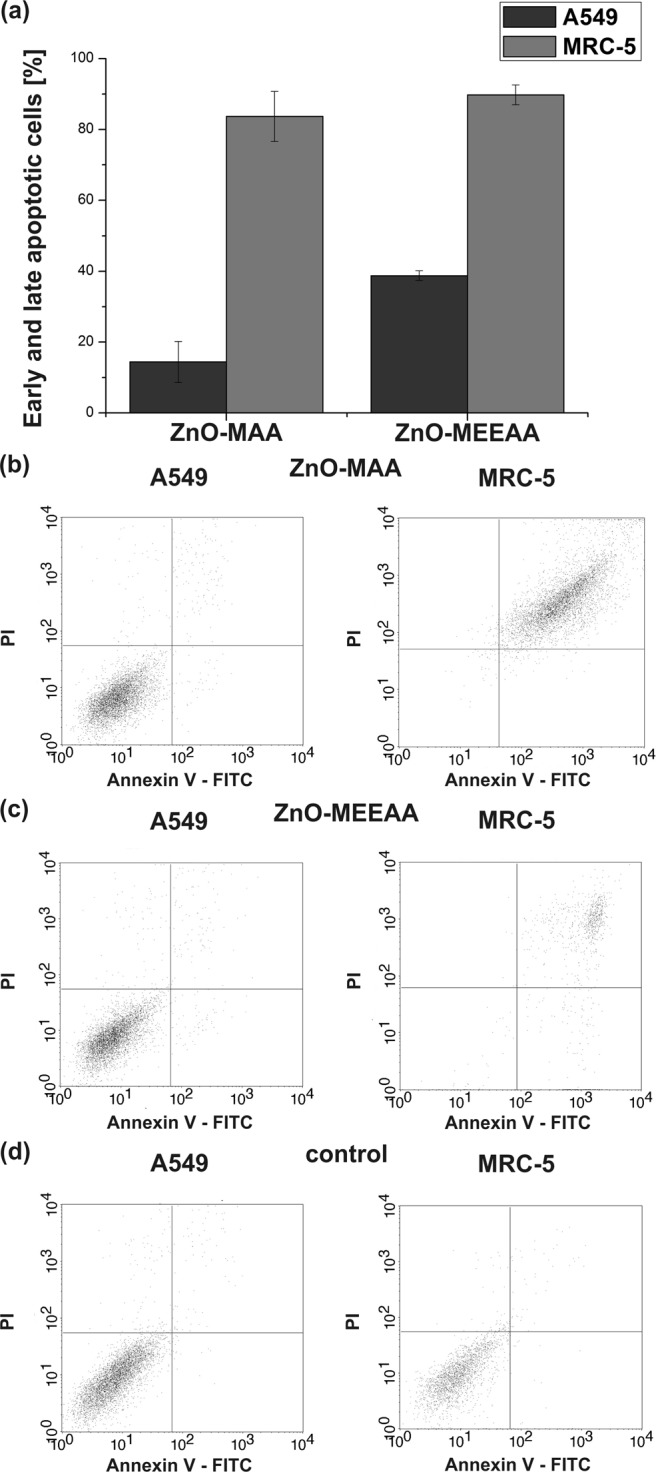
The apoptosis rate in A549 and MRC-5 cells treated with ZnO-MAA (**b**), ZnO-MEEAA (**c**) and untreated with ZnO-AAA NCs cells (**d**) detected using flow cytometry. The percentage of early and late apoptotic cells is presented in graph (**a**). Flow charts: lower right quadrant, Annexin V positive and PI negative cells indicates early apoptotic cells; upper right quadrant, Annexin V and PI-positive cells represents necrotic or late apoptotic cells.

To investigate if the cell apoptosis could result from the interaction with ZnO-MAA and ZnO-MEEAA NCs, the proportion of apoptotic cells was determined using Flow Cytometry with double staining of cell cultures with PI and Annexin V – FITC. The evaluated cell apoptosis induced by ZnO-MAA and ZnO-MEEAA NCs in A549 and MRC-5 cells is shown in Fig. 6. We found that the ZnO NCs coated by carboxylates bearing the shortest and the longest ether tail induced significant MRC-5 cell apoptosis (84% and 90% apoptotic cells, respectively) compared to A549 cells (14% and 39% apoptotic cells, respectively) (Table S2 in the SI). Generally, the ratio of apoptosis cells was higher for ZnO-MEEAA NCs (Fig. 6), which was in good correlation with the cell metabolic activity data.

## Conclusion

In the foregoing account, we presented the results describing the organometallic synthesis of a series of colloidal ZnO NCs coated with various monoanionic alkoxyacetate ligands with strongly anchoring the carboxylate head-group and different ether chain lengths. We proved that the basic physicochemical characteristic of alkoxyacetate ligands-coated quantum-sized ZnO crystals (including features such as inorganic core size, hydrodynamic diameter, the thickness of organic coating, surface charge, solution-stability and PL quantum yield) is determined almost exclusively by the character of the applied alkoxyacetate ligand. Both the uniformity and the size of the inorganic core of the resulting NCs are strongly affected by the tail length of the capping ligand, being smallest and narrowest for the NCs coated with the ligand with the longest ether chain. Besides, the NCs stabilized with the longest ligands are the most solution-stable over time and exhibit the highest PL quantum yield. Moreover, the studied NCs exhibit ultralong-lived electron-hole separation up to 2.4 µs and remarkably no significant differences were found in both absorption and luminescence profiles as well as in the PL decays of the analyzed nanosystems. Thus, it is reasonable to assume that the charge-carrier separation and transfer processes presumably relate to the unique inorganic core-organic shell interface of ZnO NCs engineered by the OSSOM method. Additionally, the ZnO NCs-induced cytotoxicity was determined using MTT assay in human lung cells and correlated with their structure-property relationship. Noticeably, no negative effect has been observed up to the NCs concentration of 10 µg/ml. For ZnO NCs coated by carboxylates bearing the shortest and the longest ether tail the doses above 10 µg/ml induced a significant reduction in cell viability, while for ZnO NCs capped with medium tail ligand only a minor effect, even at the concentration of 25 µg/ml, could be observed. In the ROS analysis, for all kinds of ZnO NCs, even at concentrations as high as 25 µg/ml the changes observed in ROS level were negligible, and therefore, the cytotoxicity of and cell death induced by the high concentrations of the alkoxylate ligand-coated ZnO NCs could not be considered as a ROS-dependent process. We believe that our work is an important contribution towards better understanding of the processes behind nano-specific toxicity and towards developing of the improved safe-by-design strategy for the preparation of ZnO NCs for various particle-based bio-applications.

## Experimental

### General remarks

All manipulations connected with the synthesis of organometallic precursors were conducted under dry and oxygen-free nitrogen atmosphere in carefully dried glassware using standard Schlenk techniques. All reagents were used without further purification and were purchased from commercial sources as follows: methoxyacetic acid (Sigma Aldrich), 2-(2-methoxyethoxy)acetic acid (Sigma Aldrich), 2-[2-(2-methoxyethoxy)ethoxy)acetic acid (Sigma Aldrich), Et_2_Zn (ABCR). Solvents (THF and hexane) were dried and distilled from sodium−potassium alloy and benzophenone prior to use.

### Methods

Liquid-state NMR spectra were acquired on a Varian Mercury 400 MHz spectrometer at 298 K. FTIR spectra were measured with a Bruker Vertex 80 V spectrometer. Size and shape of the ZnO NCs were examined using a C_s_-corrected Scanning Transmission Electron Microscope (HITACHI HD2700, 200 kV). For STEM measurements ZnO NCs samples were drop-cast (DMSO solution) onto 300-mesh, holey carbon-coated copper grids (Quantifoil). Size distributions of ZnO NCs were obtained by measuring at least 100 separated NCs of each sample. Powder XRD data were collected on an Empyrean diffractometer (PANalytical) by employing Ni-filtered Cu_Kα_ radiation from a copper sealed tube charged with 40 kV voltage and 40 mA current in Bragg-Brentano geometry with beam divergence of 1 deg. in the scattering plane. Diffraction patterns were measured in the range of 20–80 degrees of scattering angle by step scanning with step of 0.008 degree. Thermogravimetric analysis (TGA) was carried out using a TA Instruments Q600 under a flow of artificial air, to max 600 °C, at a heating rate of 5 °C/min (flow rate of 100 mL/min). Hydrodynamic diameters of ZnO NCs were determined by Dynamic Light Scattering (DLS) performed on a Malvern Zetasizer Nano Z. A solution of ZnO NCs in different organic solvents (THF and DMSO respectively) were filtered before the analysis through a 0.2 micron filter. Surface zeta-potential of ZnO NCs solutions in water were assessed by a Malvern Zetasizer Nano ZS at room temperature. A minimum of three measurements per sample were made. Optical absorption (UV-Vis) spectra for ZnO NCs colloidal solutions in THF and DMSO were collected on a HITACHI U-2910 spectrophotometer. A standard quartz cell (Hellma) with a 10 mm path length was used. Photoluminescence (PL) measurements were carried out using a HITACHI Fluorescence Spectrophotometer F-7000. Fluorescence quantum yields were measured using a C9920-02G absolute quantum yield (QY) measurement system from Hamamatsu. The photoluminescence (PL) decays were collected in the same manner as previously described^17^ using an Edinburgh Instruments CD900 spectrometer equipped with a diode laser EPLED 320 (40 μW at 100 kHz repetition rate) as an excitation source. The samples were excited at 320 nm and PL was collected either at 540 nm or 550 nm i.e. at the maximum of the steady-state PL emission spectrum corresponding to a particular sample. The spectra were collected at 20 nm bandpass (530–550 nm for the maximum at 540 nm and 540–560 nm for the maximum at 550 nm). Additionally, a 390 nm cut-off filter was used to eliminate the second order artifacts. Measurements were carried out with the emission monitored at a 90° angle to the excitation. The data were collected in 4096 channels with 10000 counts at the peak, and the time calibration was 2.441 ns per channel. The data were analyzed by a least squares reconvolution procedure using the software package provided by Edinburgh Instruments. Goodness of fit was judged in terms of χ^2^ value and residuals distribution. When lower than 1.3, the χ^2^ values were taken as appropriate for the kinetic model and resulting parameters.

### Synthesis of [EtZn(AAA)]-type organometallic precursors

#### Synthesis of [EtZn(MEAA)]_n_

The procedure for [EtZn(MEAA)]_n_ synthesis and its transformation to ZnO-MEAA NCs as well as ZnO-MEAA NCs properties were reported previously^17^;

#### Synthesis of [EtZn(MAA)]_n_

An appropriate amount of methoxyacetic acid (0.180 g, 2 mmol) was dissolved in THF (10 mL) and cooled to -78 °C under N_2_ atmosphere. Then 2 mmol of Et_2_Zn (0.95 mL of a 2.10 M solution in hexane) was added dropwise under vigorous stirring. After few minutes the reaction was gradually warmed up to room temperature. The solvent was removed from the resulting reaction mixture under reduced pressure to give a crude product [EtZn(MAA)]_n_ at almost quantitatively yield. ^1^H NMR (400 MHz, C_6_D_6_, 25 °C, δ): 0.72 (q, 2H, CH_3_-*CH*_*2*_-Zn); 1.63 (t, 3H, *CH*_*3*_-CH_2_-Zn); 3.00 (s, 3H, *CH*_*3*_*O*-CH_2_-COO); 3.80 (s, 2H, CH_3_O-*CH*_*2*_-COO) ppm; IR (ATR) ṽ = 3003 (vw), 2935 (w), 2889 (w), 2852 (w), 2834 (vw), 2808 (vw), 1606 (vs), 1584 (vs), 1468 (w), 1442 (s), 1426 (m), 1371 (vw), 1339 (m), 1322 (m), 1232 (vw), 1203 (m), 1104 (vs), 1019 (vw), 976 (s), 948 (m), 923 (w), 728 (w), 609 (m), 591 (w), 538 (m), 516 (s), 428 (w), 419 (m), 416 (m), 4002 (w) cm^−1^;

#### Synthesis of [EtZn(MEEAA)]_n_

The procedure was similar to that described for [EtZn(MAA)]_n_, using 2 mmol of Et_2_Zn (0.95 mL of a 2.10 M solution in hexane) and 2 mmol (0.356 g) of 2-[2-(2-methoxyethoxy)ethoxy]acetic acid. The solvent was removed from the resulting reaction mixture under reduced pressure and a crude product [EtZn(MEEAA)]_n_ was obtained almost quantitatively. ^1^H NMR (400 MHz, C_6_D_6_, 25 °C, δ): 0.71 (q, 2H, CH_3_-*CH*_*2*_-Zn); 1.65 (t, 3H, *CH*_*3*_-CH_2_-Zn); 3.12 (s, 3H, *CH*_*3*_O-CH_2_-CH_2_); 3.33 (m, 2H, O-CH_2_-*CH*_*2*_-OCH_2_-COO); 3.40 (m, 2H, O-*CH*_*2*_-CH_2_-OCH_2_-COO); 3.43 (m, 2H, CH_3_O-CH_2_-*CH*_*2*_); 3.61 (s, 2H, CH_3_O-*CH*_*2*_-CH_2_); 4.22 (s, 2H, CH_2_O-*CH*_*2*_-COO) ppm; IR (ATR) ṽ = 2924 (w), 2877 (w), 2820 (vw), 1601 (vs), 1587 (vs), 1430 (m), 1417 (m), 1370 (vw), 1354 (vw), 1327 (m), 1294 (w), 1248 (w), 1199 (w), 1101 (vs), 1028 (w), 987 (w), 951 (w), 930 (w), 890 (w), 850 (w), 722 (w), 603 (w), 512 (m) cm^−1^.

### General procedure of ZnO-AAA NCs preparation

ZnO-AAA NCs were prepared in one-pot two step synthesis using organometallic OSSOM approach. In a general synthesis, 2 mmol of Et_2_Zn (0.95 mL of a 2.1 M solution in hexane) was added to a stirred solution of the selected organic carboxylate pro-ligand (2 mmol) in THF (10 ml) at −78 °C. Then, the reaction mixture was warmed up to room temperature and stirred vigorously for additional 6 h. The preparation of a variety of ZnO NCs coated by selected ligands was realized through the exposition of the organometallic precursor solution in THF to the air at room temperature for minimum 5–7 days. For [EtZn(MAA)]_n_ precursor, the reaction mixture after the exposition to the air forms a precipitate that is not soluble in common organic solvents (i.e. THF, hexane, toluene, etc.) Then white, solid sediment was removed by filtration process. For the remaining ([EtZn(MEAA’)] and [EtZn(MEEAA’)]) reaction mixture is clear and colorless. The overall synthesis requires several days, but advantageously, it yields multigram quantities of stable and redispersible ZnO-AAA’ NCs. The NCs were characterized by FTIR analysis before purification process. **ZnO-MAA’:** IR (ATR) ṽ = 3307 (w), 2957 (vw), 2884 (w), 1764 (vw), 1664 (vw), 1601 (m), 1579 (m), 1454 (vw), 1439 (w), 1427 (w), 1419 (w), 1367 (vw), 1345 (w), 1322 (vw), 1237 (vw), 1191 (w), 1119 (vw), 1110 (w), 1095 (w), 1069 (s), 1035 (s), 960 (m), 923 (s), 882 (w), 847 (w), 743 (w), 730 (w), 451 (vs), 423 (vs) cm^−1^; **ZnO-MEEAA’:** IR (ATR) ṽ = 3322 (vw), 2941 (w), 2878 (w), 2829 (vw), 1773 (vw), 1589 (vs), 1417 (s), 1367 (w), 1355 (w), 1329 (m), 1297 (w), 1260 (w), 1197 (w), 1098 (vs), 1066 (vs), 1034 (vs), 989 (m), 958 (s), 925 (s), 887 (w), 849 (m), 803 (w), 722 (m), 597 (m) cm^−1^.

### Purification process

As-synthesized ZnO-AAA’ NCs were separated from the resulting THF solution using hexane and rewashed two times with THF. Then, ZnO-AAA NCs were dried under vacuum and used for characterization. Infrared spectra obtained after purification process are described below. It is worth noting that the traces of a proper parent protonated AAA-H are no more observed after purification (Fig. S3 in the SI). **ZnO-MAA:** IR (ATR) ṽ = 3409 (vw), 2934 (vw), 2827 (vw), 1599 (w), 1580 (w), 1455 (vw), 1441 (w), 1428 (w), 1419 (w), 1345 (vw), 1200 (vw), 1110 (w), 1097 (w), 1017 (vw), 986 (vw), 978 (vw), 945 (vw), 919 (vw), 727 (vw), 424 (vs) cm^−1^; **ZnO-MEEAA:** IR (ATR) ṽ = 3367 (vw), 2975 (vw), 2925 (vw), 2874 (w), 2824 (vw), 1588 (vs), 1417 (m), 1354 (vw), 1328 (m), 1297 (w), 1248 (vw), 1200 (vw), 1100 (vs), 1027 (w), 1007 (w), 959 (w), 928 (w), 888 (w), 850 (w), 720 (m), 599 (w), 456 (s) cm^−1^.

### Cell line and cell culture

A cancer human alveolar epithelial cells (A549) and human fetal lung fibroblast cells (MRC-5) were purchased from the American Type Culture Collection (ATCC). Both A549 and MRC-5 cells were cultured in MEME (Sigma Aldrich) supplemented with 10% (v/v) Fetal Bovine Serum (Gibco), 1% (v/v) 100 U/mL penicillin and 100 mg/mL streptomycin (Sigma Aldrich), 1% (v/v) 200 mg/L-Glutamine (Sigma Aldrich) and 1% (v/v) Nonessential Amino Acids (Sigma Aldrich, only for MRC-5 line). Cells were maintained in a humidified atmosphere at 37 °C and 5% CO_2_ (HeraCell). The culture medium was changed every 2-3 days and passage protocol was performed by trypsinization when cells reached 70–90% confluence. In general biological studies were performed according to standard procedures and the ZnO NCs samples were prepared as recently described^17^.

### MTT viability assay

Cytotoxicity of the ZnO-AAA NCs was assessed using standard MTT assay (Sigma Aldrich) according to the manufacturer’s protocol. Briefly, cells were seeded into a 96-well plates at a density of 10^4^ cells per well (note that such density provides an appropriate degree of confluence of the cells of approximately 70% when treating cells with NCs^77,78^). Then, after incubation at 37 °C in a humidified atmosphere with 5% CO_2_ for 24 h, cells were treated with a series of concentrations of ZnO NCs. The ZnO-AAA NCs (10 mg/mL stock solution in DMSO) were suspended in serum-free medium to the final concentrations of 5, 7.5, 10, 15, 20 and 25 µg/mL (note that samples contained safe for the cells percentage limit of DMSO which have been recently confirmed in the corresponding cell culture^17^) and were carefully sonicated for 15 min in order to limit agglomeration process. Subsequently, 100 μL of each ZnO-AAA NCs suspensions were added to the A549 and MRC-5 cells and incubated for 24 h. The absorbance of purple formazan was measured directly in the wells at 570 nm using microplate reader (Cytation 3, BioTek). The relative cell viability was determined as a percentage of the control (untreated with ZnO NCs cells incubated in fresh serum-free medium). Results were expressed as the mean ± standard deviation (SD) from at least four individual experiments with six replicate wells. Statistical analyses were performed by one-way analysis of variance (ANOVA) followed by Tukey’s post hoc test. A p value less than 0.05 was considered statistically significant. Additionally, microscopic observation (Olympus IX71) of cell culture was conducted. For this purpose cells were stained with Calcein-AM and Propidium Iodide (Sigma Aldrich) after treatment with ZnO-AAA NCs. The cellSens image analysis software (Olympus) was used for data acquisition and image analysis.

### Measurement of intracellular ROS

In order to delineate the nano-specific toxicity of ZnO-AAA NCs, the generation of intracellular ROS was examined using cell permeable fluorogenic probe 2,7-dichlorofluorescein diacetate (DCFH-DA, Sigma). A549 and MRC-5 cells were treated with 5, 15, 25 µg/mL ZnO NCs for 24 h. Then, cells were washed with PBS prior to incubation with 40 μM DCFH-DA in dark in serum-free cultured medium and incubated at 37 °C for 30 min. The fluorescence intensity of DCF was measured using the microplate reader at λ_ex_ 485 nm and λ_em_ 530 nm. The DCF concentration in untreated cells was used as a control.

### Flow cytometry

The next stage of research was to investigate whether the cytotoxic effect of the ZnO NCs is associated with the cell apoptosis. Briefly, the A549 and MRC-5 cells were grown to 80–90% confluence in a 24 multi-well plates in three individual experiments. Base on the MTT evaluation, cells were treated with 25 µg/mL ZnO-MAA and ZnO-MEEAA NCs for 24 h (note that comparable concentration of ZnO-MEAA NCs exhibit minor toxicological effects both for MRC-5 cells and A549 cells). After exposure, cells were collected from the wells and stained with Propidium Iodide (PI) and Annexin V – FITC (Annexin V FITC apoptosis detection kit, BectonDickinson, USA) and then analyzed using a flow cytometry (BectonDickinson, USA). Each experiment was performed three times and repeated for at least three individual experiments.

## Supplementary information

Supporting Information for: ZnO nanocrystals derived from organometallic approach: Delineating the role of organic ligand shell on physicochemical properties and nano-specific toxicity
