# Supplementary material for: ZnO nanocrystals derived from organometallic approach: Delineating the role of organic ligand shell on physicochemical properties and nano-specific toxicity
Source: Sci Rep. 2019 Dec 2;9:18071. doi: 10.1038/s41598-019-54509-z (PMC6889378; doi:10.1038/s41598-019-54509-z)
Supplement: Supplementary file 1 — Supporting Information for: ZnO nanocrystals derived from organometallic approach: Delineating the role of organic ligand shell on physicochemical properties and nano-specific toxicity [file 41598_2019_54509_MOESM1_ESM.pdf]

Supporting Information for:

ZnO nanocrystals derived from organometallic approach: Delineating the role of organic ligand shell on physicochemical properties and nano-specific toxicity

Małgorzata Wolska-Pietkiewicz*[1], Katarzyna Tokarska[1,2], Anna Wojewódzka[1], Katarzyna Wójcik[3], Elżbieta Chwojnowska[3], Justyna Grzonka[3,4], Piotr J. Cywiński[3], Michał Chudy*[1] and Janusz Lewiński*[1, 3]

[1] Faculty of Chemistry, Warsaw University of Technology, Noakowskiego 3, 00-664 Warsaw, Poland
[2] Centre for Advanced Materials and Technologies CEZAMAT Warsaw University of Technology, Poleczki 19, 02-822 Warsaw, Poland
[3] Institute of Physical Chemistry, Polish Academy of Sciences, Kasprzaka 44/52, 01-224 Warsaw, Poland
[4] Faculty of Materials Science and Engineering, Warsaw University of Technology, Wołoska 141, 02-507 Warsaw, Poland

Table of contents

1. The estimation of the NCs size from the absorption measurement. S2
2. Powder X-ray diffraction analysis S3
3. FTIR analysis S4
4. Thermogravimetric analysis S6
5. Spectroscopic methods S6
6. Determination of extinction coefficient (μ) for ZnO-AAA NCs S7
7. Stability measurements S8
8. Photoluminescence decay measurements for ZnO NCs S10
9. Flow cytometry S11
10. References S11

Throughout the main text and the Supporting Information the following nomenclature is used:

AAA-H – alkoxyacetic acid
AAA - alkoxyacetate moiety
MAA-H – methoxyacetic acid
MEAA-H – 2-(2-methoxyethoxy)acetic acid
MEEAA-H – 2-[2-(2-methoxyethoxy)ethoxy]acetic acid

ZnO-AAA NCs – as-synthetized zinc oxide nanocrystals (ZnO NCs) coated by selected monoanionic carboxylate: MAA, MEAA, MEEAA, respectively.

1. The estimation of the NCs size from the absorption measurement.

The Brus formula can be used to describe the emission energy of semiconductor NCs (including ZnO NCs) in terms of the band gap energy Eg.[1,2]. We used it to estimate the radius of a ZnO-AAA NCs from experimentally determined optical parameters. The size was compared with sizes estimated using other techniques (DLS, TEM, PXRD).

Equation S1. Brus formula to estimate NCs radius from experimentally determined parameters, where *Eg* – cluster band gap energy calculated from absorption spectrum, $Eg^{(s)}$ – solid state band gap energy, R – NCs radius.

$$E_g = E_g^{(s)} + \left(\frac{\pi^2}{R^2}\right) - \left(\frac{3.6}{R}\right)$$

2. Powder X-ray diffraction analysis

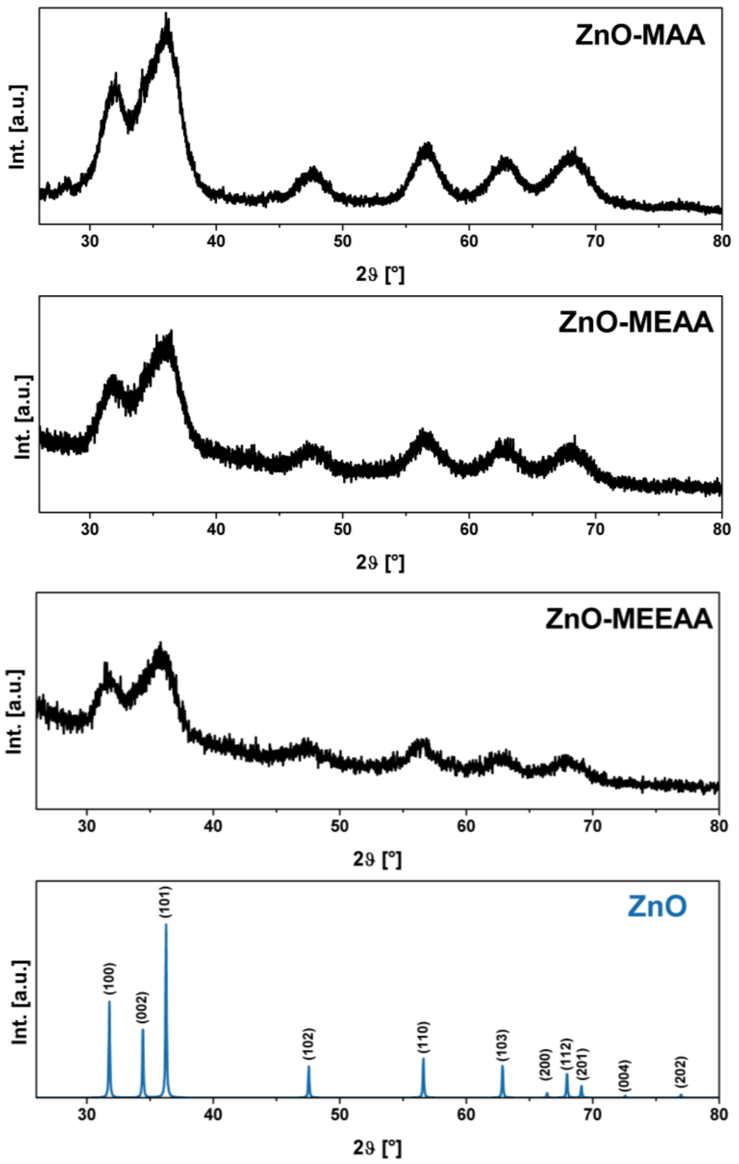

Figure S1. Powder X-ray diffraction pattern of ZnO-MAA, ZnO-MEAA and ZnO-MEEAA NCs confirming ZnO wurtzite crystalline structure.

3. FTIR analysis

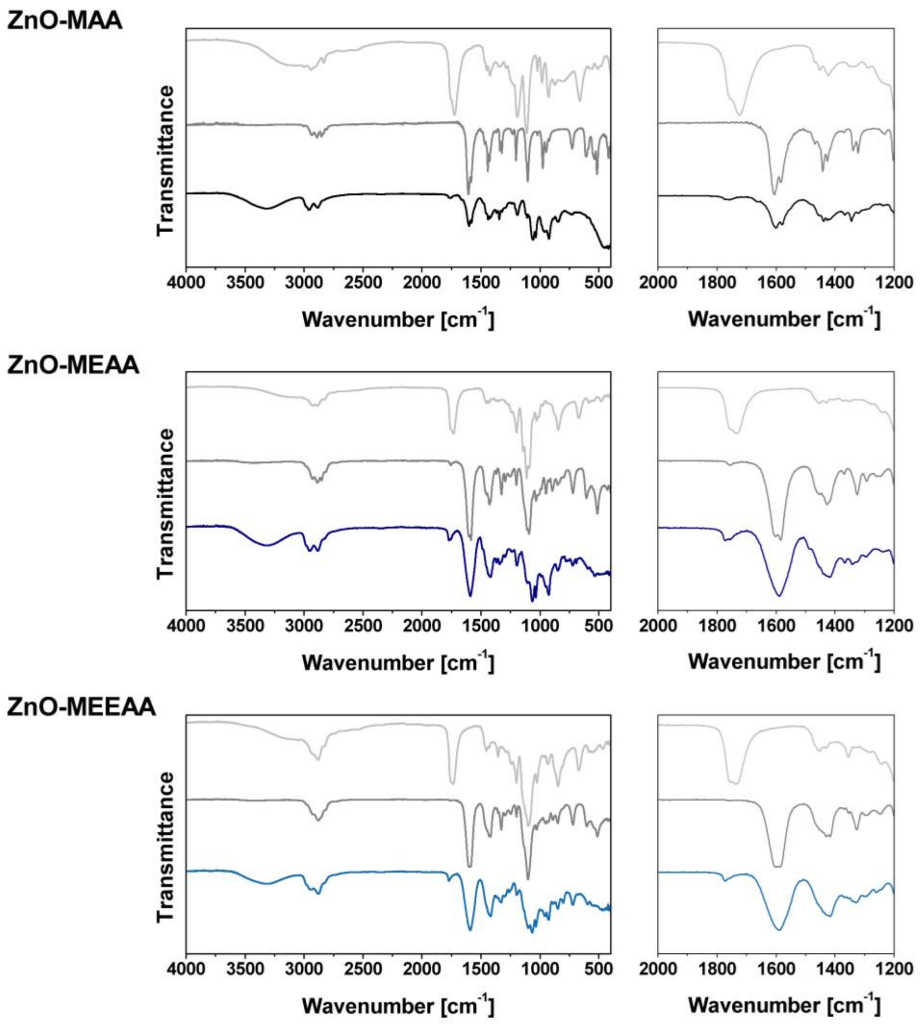

Figure S2. FTIR spectra of pure pro-ligands (grey line), organometallic [EtZn(AAA)]-type precursors (dark grey line) and as-prepared ZnO NCs, i.e. ZnO-MAA (black line), ZnO-MEA (navy blue line) and ZnO-MEEAA (blue line) NCs.

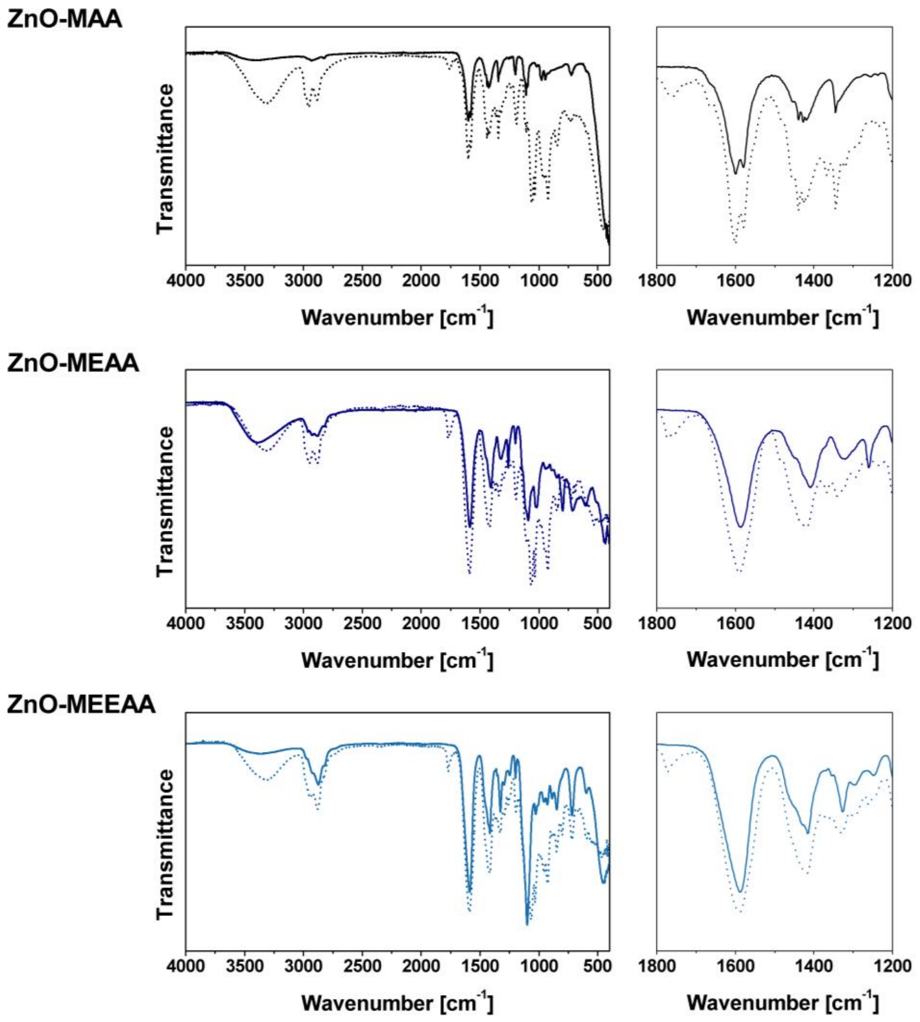

Figure S3. FTIR spectra of ZnO-AAA NCs before (dotted line) and after purification process (solid line).

4. Thermogravimetric analysis

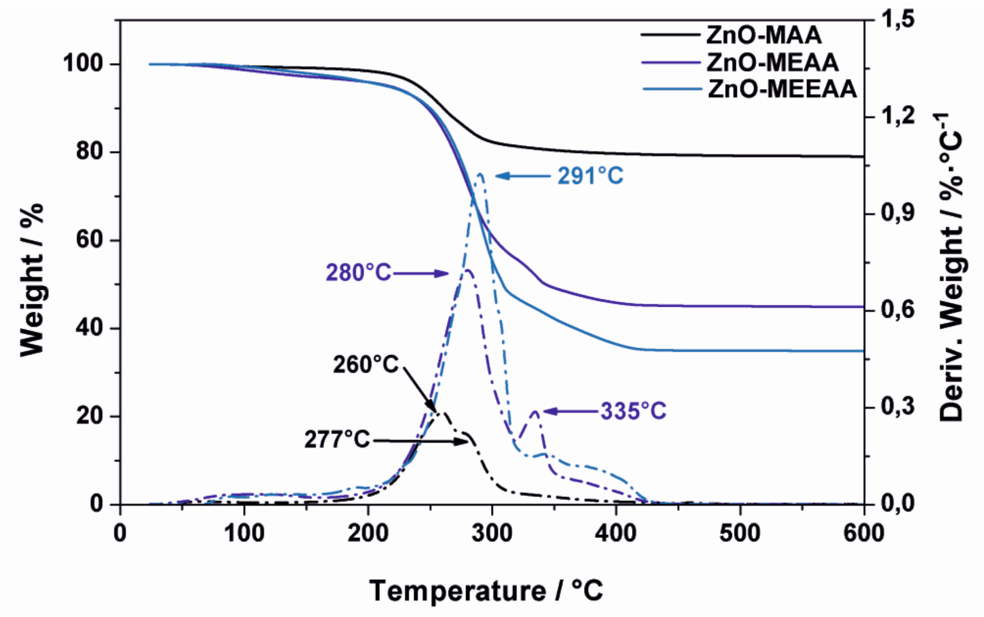

Figure S4. The TGA and derivative thermogravimetric analysis (DTG) traces showing the decomposition of ZnO-MAA (black), ZnO-MEAA (navy blue) and ZnO-MEEAA NCs (blue) in an air atmosphere.

5. Spectroscopic methods

a) b)

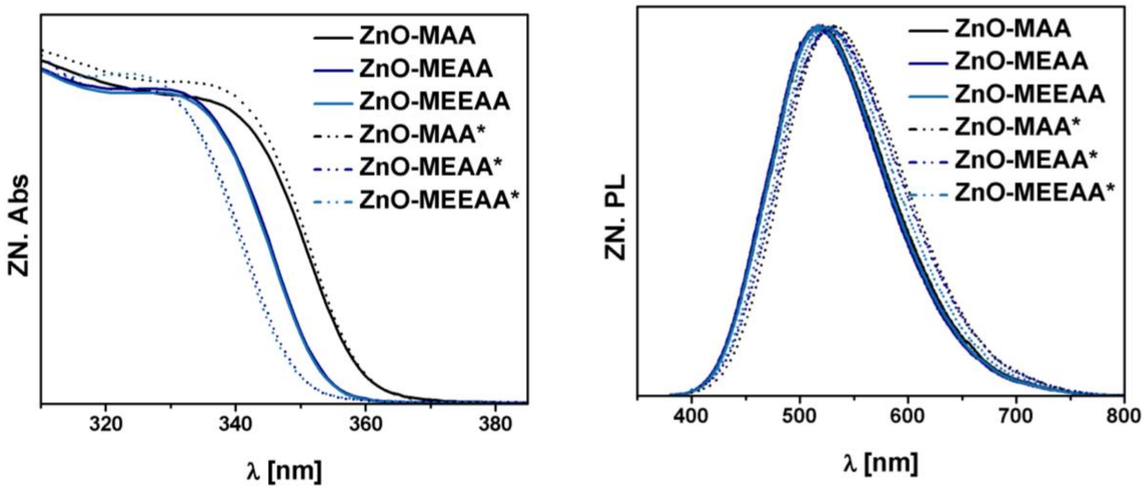

Figure S5. a) Absorption and b) emission spectra of ZnO-AAA NCs collected in THF (solid line) and DMSO (dotted line).

6. Determination of extinction coefficient (μ) for ZnO-AAA NCs

The measurements were recorded on a UV-VIS absorption spectrometer Lambda 35 + PTP A (PerkinElmer) in the spectral range of 300nm to 400nm._The mass extinction coefficient (μ) is a measure that determines how strongly a substance absorbs light at a given wavelength, per unit mass. During this study, mass extinction coefficients were calculated from Beer-Lambert Law $\boldsymbol{A = \varepsilon c l}$. The Beer-Lambert Law states that absorbance of a sample depends on the molar concentration, light path length in centimeters and molar extinction coefficient for the dissolved substance at the specified wavelength (λ). In our case, instead of molar extinction coefficient, we introduced mass attenuation coefficient and mass concentration (in mg/mL or μm/mL) instead of molar concentration and that led to a modified Beer–Lambert Law ($\boldsymbol{A =}$ μ$\boldsymbol{cl}$.). When presenting absorbance in the dependence on the mass, the mass extinction coefficient can be determined from the slope of this dependence. All the obtained results are presented below.

ZnO-MAA

a)

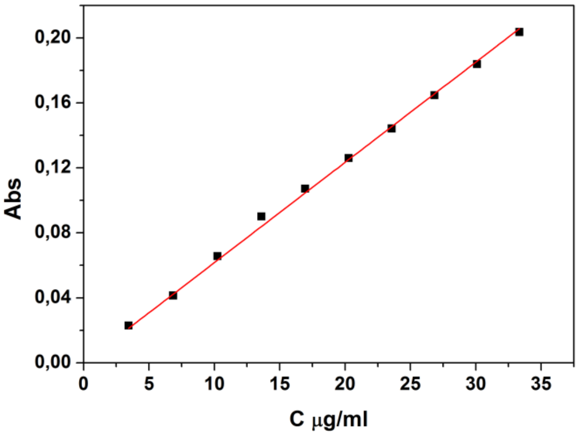

μ = 6.10 ± 0.04 mL·mg^{-1}·cm^{-1}
R^2 = 0.99956

b)

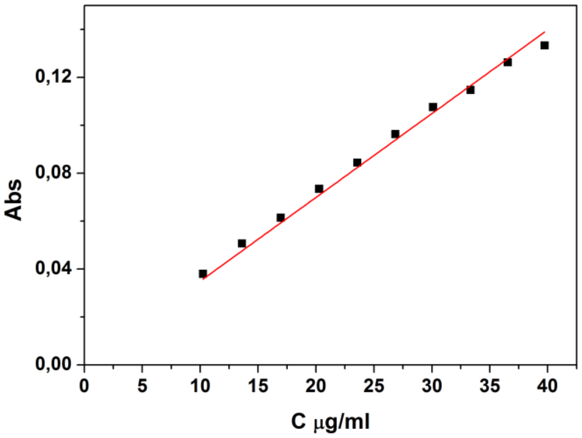

μ = 3.50 ± 0.03 mL·mg^{-1}·cm^{-1}
R^2 = 0.99901

ZnO-MEAA

c)

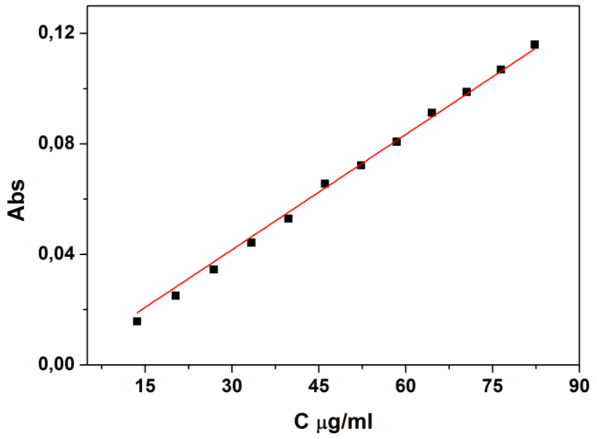

μ = 1.39 ± 0.01 mL·mg^{-1}·cm^{-1}
R^2 = 0.99924

d)

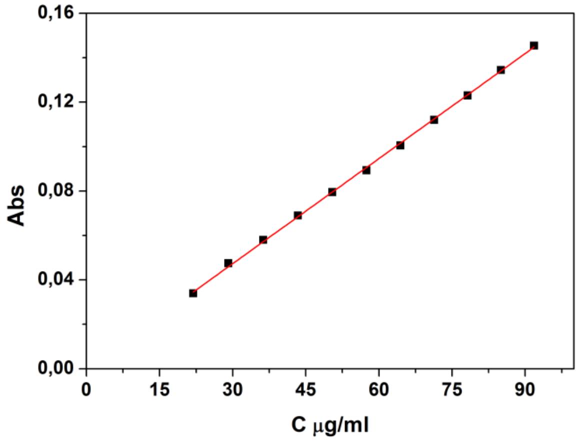

μ = 1.58 ± 0.004 mL·mg^{-1}·cm^{-1}
R^2 = 0.99992

ZnO-MEEAA

e)

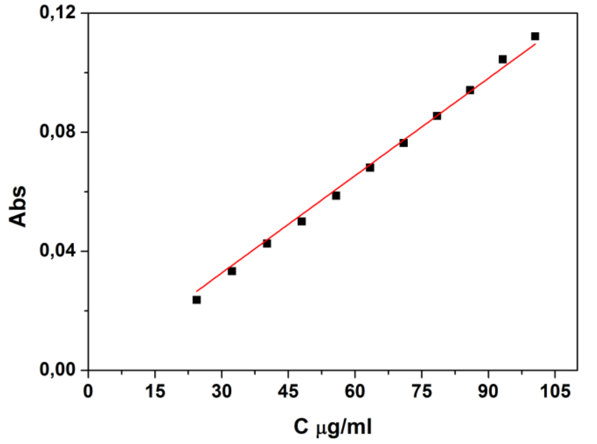

μ = 1.09 ± 0.01 mL·mg^{-1}·cm^{-1}
R^2 = 0.99925

f)

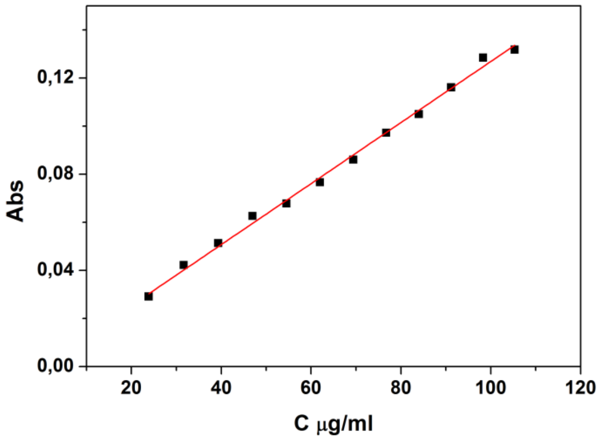

μ = 1.27 ± 0.01 mL·mg^{-1}·cm^{-1}
R^2 = 0.99946

Figure S6. Determination of extinction coefficient (μ) for ZnO AAA NCs dissolved in THF (a, c, e) or in DMSO (b, d, f).

7. Stability measurements

In order to determine long-term stability for synthesized ZnO-AAA NCs, spectroscopic shelf-life studies were performed in THF and in DMSO. Closed samples, prepared by dissolving ZnO-MAA NCs, ZnO-MEAA or ZnO-MEEAA NCs in chosen solvent were measured day by day up to 35, 66 and 75 days, respectively. The results for ZnO-AAA NCs are presented in the Figure S7.

a)

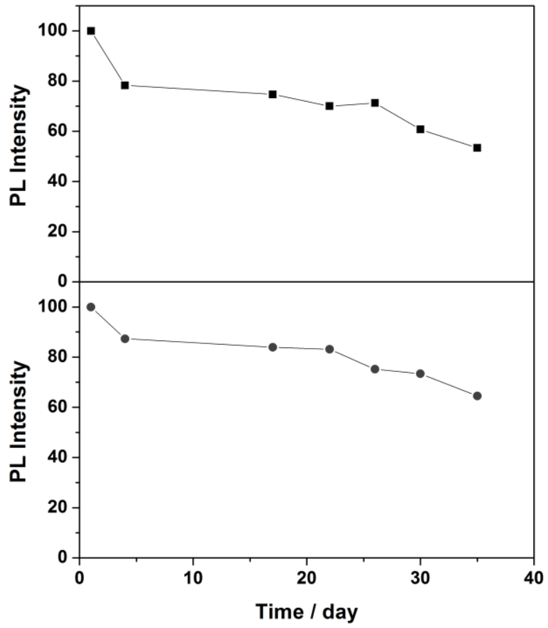

b)

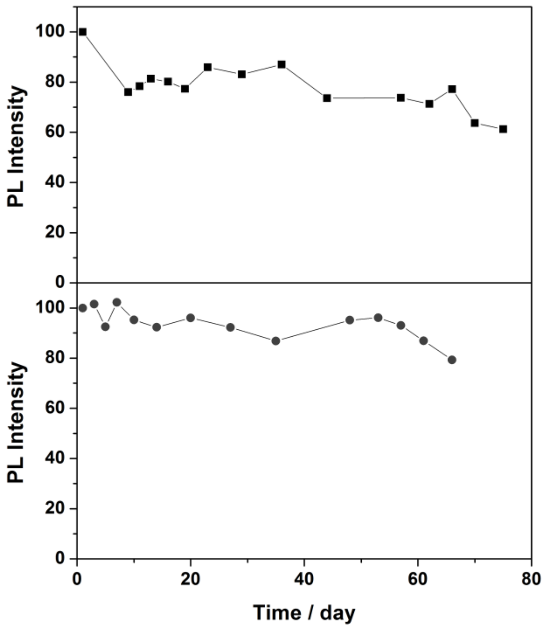

c)

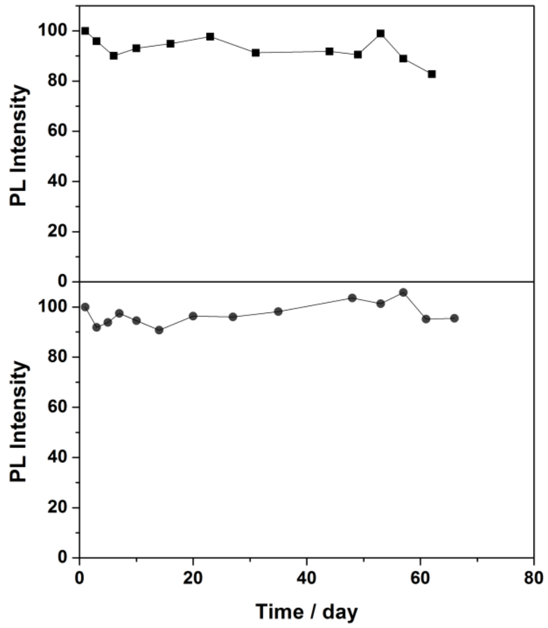

d)

	THF	DMSO
**MAA**	**46.6**	**35.4**
**MEAA**	**38.7**	**20.7**
**MEEAA**	**17.2**	**4.5**

***the values(%) relate to the decline of optical properties**

Figure S7. PL intensity (%) measured for a) ZnO-MAA b) ZnO-MEAA and c) ZnO-MEEAA NCs in both THF (black squares) and DMSO (dark grey circle) solution; d) correlation between decrease of PL intensity and organic ligand structure which strongly affect the stability of resulting NCs in different solvents.

8. Photoluminescence decay measurements for ZnO NCs

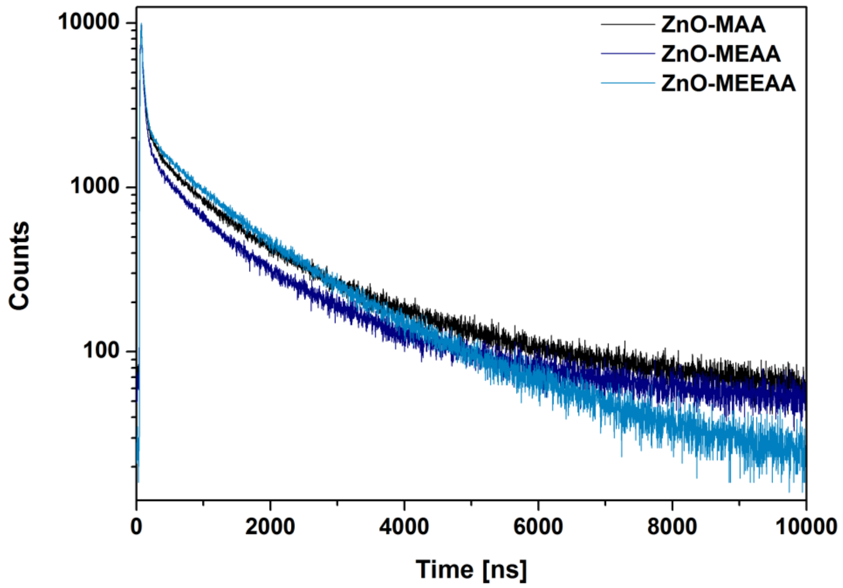

Figure S8. Photoluminescence decays taken for ZnO-AAA NCs.

Table S1. The photoluminescence decay times and corresponding contributions fitted using 4-exponential function (Eq.S2).

Sample ID	Photoluminescence decay time	Contribution [%]
**ZnO-MAA**	τ_1= (27.77 ± 0.60) ns<br>τ_2= (120.63 ± 8.07) ns<br>τ_3= (777.26 ± 24.19) ns<br>τ_4= (2.36 ± 0.06) µs	68.95<br>13.12<br>11.95<br>5.99
**ZnO-MEAA**	τ_1= (26.29 ± 0.72) ns<br>τ_2= (93.88 ± 7.07) ns<br>τ_3= (701.67 ± 19.46) ns<br>τ_4= (2.14 ± 0.06) µs	68.85<br>14.70<br>11.41<br>5.04
**ZnO-MEEAA**	τ_1= (34.11 ± 0.66) ns<br>τ_2= (148.59 ± 13.61) ns<br>τ_3= (972.39 ± 30.80) ns<br>τ_4= (2.43 ± 0.10) µs	71.18<br>7.90<br>15.17<br>5.75

Equation S2. The four-exponential function used to fit the PL decays for ZnO-AAA NCs.

$$I(t) = A_1 \exp\left(-\frac{t}{\tau_1}\right) + A_2 \exp\left(-\frac{t}{\tau_2}\right) + A_3 \exp\left(-\frac{t}{\tau_3}\right) + A_4 \exp\left(-\frac{t}{\tau_4}\right)$$

9. Flow cytometry

Table S2. Number of apoptotic cells of A549 and MRC-5 cells after a 24 h incubation with ZnO AAA NCs.

	apoptotic A549 cells/%	***apoptotic MRC-5 cells/%***
ZnO-MAA	14.41±5.81	83.69±7.07
ZnO-MEEAA	38.75±1.40	89.75±2.79

10. References

(1) Brus, L. Electronic wave-functions in semiconductor clusters – experiment and theory. *J. Phys. Chem*. **90**, 2555–2560 (1986).

(2) Monticone, S.; Tufeu, R. & Kanaev, A. V. Complex nature of the UV and visible fluorescence of Colloidal ZnO nanoparticles. *J. Phys. Chem. B* **102**, 2854–2862 (1998).
